# Longitudinal Survey of Carotenoids in Human Milk from Urban Cohorts in China, Mexico, and the USA

**DOI:** 10.1371/journal.pone.0127729

**Published:** 2015-06-10

**Authors:** Tristan E. Lipkie, Ardythe L. Morrow, Zeina E. Jouni, Robert J. McMahon, Mario G. Ferruzzi

**Affiliations:** 1 Department of Food Science, Purdue University, West Lafayette, Indiana, United States of America; 2 Department of Pediatrics, Cincinnati Children’s Hospital, Cincinnati, Ohio, United States of America; 3 Global Discovery and Analytical Science, Mead Johnson Nutrition Institute, Evansville, Indiana, United States of America; 4 Department of Nutrition Science, Purdue University, West Lafayette, Indiana, United States of America; USDA/ARS, UNITED STATES

## Abstract

Emerging evidence indicates that carotenoids may have particular roles in infant nutrition and development, yet data on the profile and bioavailability of carotenoids from human milk remain sparse. Milk was longitudinally collected at 2, 4, 13, and 26 weeks postpartum from twenty mothers each in China, Mexico, and the USA in the Global Exploration of Human Milk Study (n = 60 donors, n = 240 samples). Maternal and neonatal plasma was analyzed for carotenoids from the USA cohort at 4 weeks postpartum. Carotenoids were analyzed by HPLC and total lipids by Creamatocrit. Across all countries and lactation stages, the top four carotenoids were lutein (median 114.4 nmol/L), β-carotene (49.4 nmol/L), β-cryptoxanthin (33.8 nmol/L), and lycopene (33.7 nmol/L). Non-provitamin A carotenoids (nmol/L) and total lipids (g/L) decreased (p<0.05) with increasing lactation stage, except the provitamin A carotenoids α- and β-cryptoxanthin and β-carotene did not significantly change (p>0.05) with lactation stage. Total carotenoid content and lutein content were greatest from China, yet lycopene was lowest from China (p<0.0001). Lutein, β-cryptoxanthin, and β-carotene, and lycopene concentrations in milk were significantly correlated to maternal plasma and neonatal plasma concentrations (p<0.05), with the exception that lycopene was not significantly associated between human milk and neonatal plasma (p>0.3). This enhanced understanding of neonatal exposure to carotenoids during development may help guide dietary recommendations and design of human milk mimetics.

## Introduction

**Fig 1:**
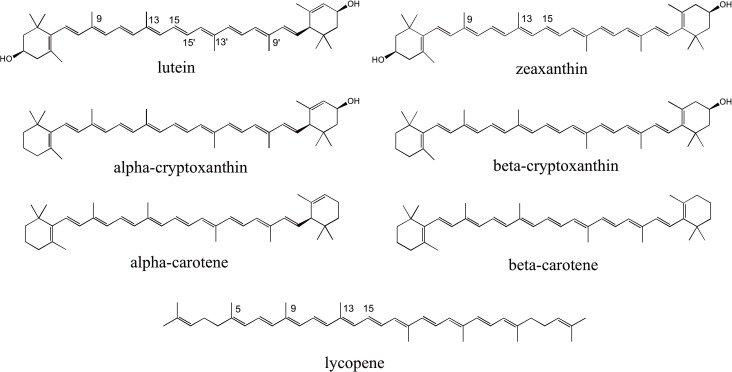
Carotenoid species identified in milk. C atoms are labeled to indicate positions of *cis-* isomers.

Human breast milk is the preferred sole source of nutrition for infants through 6 months of age due to epidemiological evidence for reduced risk of disease [1]. Milk is a complete source of nutrition, containing macronutrients and micronutrients in addition to antibodies, growth factors, and bioactive components such as lactoferrin, oligosaccharides and phytochemicals including carotenoids (see Fig 1) [2, 3]. While carotenoids are not currently considered essential nutrients besides the provitamin A activity of some, evidence suggests carotenoids may have particular roles in infant development and nutrition. Non-provitamin A carotenoids lutein and zeaxanthin protect against light stress and oxidation in the retinal pigment epithelium [4], and increased risk of retinopathy of prematurity in preterm infants is associated with low serum lutein + zeaxanthin and non-detectable macular pigment optical density [5]. Provitamin A activity of β-carotene, α-carotene, β-cryptoxanthin, and perhaps α-cryptoxanthin may be particularly important for the mother and infant. Vitamin A is required for visual and immune development [6], and evidence suggests provitamin A carotenoids are an important source of vitamin A in developing tissues [7]. Lycopene may also play a role in immune development [8] and protection against inflammatory diseases [9].

Milk composition fluctuates with lactation stage and is regulated by secretion and synthesis of components by mammary alveolar epithelial cells. At birth, colostrum is rich in lactoglobulins and other bioactive peptides. Mature milk composition is indicated around 5 days post parturition by changes such as increase in lactose and lipids, and is relatively stable through 6 months [10]. Milk production and intake averages 750–800 mL/day (30 g lipid/day) by 4 weeks, but variation between infants can range 400–1200 mL/day [10]. Lipid content of hind milk is greater than foremilk, and carotenoid concentration similarly increases as the breast is emptied [11]. Since breast fullness and completeness of milk expression influence lipid and carotenoid composition, variability of carotenoid composition is reduced when reported with respect to total lipid content [12].

Carotenoids have been observed to follow opposite trends from total lipids as lactation proceeds, suggesting that some component of carotenoid transfer follows a different mechanism from bulk lipids (triglycerides, phospholids) [13]. Milk components are secreted by one of five pathways: membrane route, Golgi route, transcytosis, paracellular, or milk fat route [14, 15].

Carotenoids are transported to the epithelial surface via lipoproteins. Carotenes and lycopene associate mostly with VLDL/LDL, while lutein and zeaxanthin are equally distributed between LDL and HDL [13, 16]. After release by lipoprotein lipase, carotenoids are likely transferred into mammary alveolar epithelial cells by fatty acid transporter and cluster determinant 36 (CD36). Lipids accumulate into droplets at the apical membrane surface, and are extruded into milk packaged within the milk fat globule membrane [14].

The composition of human milk may be the best guide for development of dietary recommendations and phytochemical fortified infant formula due to lack of balance studies to determine optimal intake [17]. While milk macronutrient composition tends to be independent of maternal dietary intake [10, 15], carotenoid content of milk is associated with maternal diet [18] and plasma carotenoid/ vitamin A status [13, 19]. Since carotenoid content of breast milk varies by country due to carotenoids in the regional dietary pattern, reference values for individual countries are needed [18]. The aims of this study were to (1) expand on previous knowledge of carotenoid profiles by collecting longitudinal carotenoid profiles of human milk in select countries, and (2) develop associations of carotenoid transfer from maternal plasma to milk to the infant. We report here the carotenoid profiles of human milk from the cohorts in the Global Exploration of Human Milk study [20].

## Materials and Methods

### Sample information

All mothers provided written informed consent and this study was approved by the Institutional Review Boards of Cincinnati Childrens Hospital Medical Center, the National Institute of Medical Sciences and Nutrition in Mexico City, and Shanghai Childrens Hospital of Fudan University. Longitudinal samples were collected at 2, 4, 13, and 26 weeks postpartum in China (Shanghai), Mexico (Mexico City), and USA (Cincinnati) as part of the Global Exploration of Human Milk Study with approval as previously described [20]. Demographics and delivery characteristics are reported in Table 1, and additional details were reported previously [20].Inclusion criteria included mother’s age 18–49 years, singleton birth, infant ≥ 2500 g, infant gestational age ≥37 weeks, live within 25 mile radius of Hospital, intention to breastfeed ≥3 months, intention to provide ≥75% breast milk, and infant born with no impediments to breastfeeding. A weekly questionnaire was administered by phone in which questions about infant feeding and supplemental foods were asked.

**Table 1:** Demographic characteristics of human milk donors.

Characteristic	Shanghai (n = 20)	Cincinnati (n = 20)	Mexico City (n = 20)
Prepregnancy BMI, median (range)	20.4 (17.8, 25.4)	26.0 (21.0, 38.4)	23.5 (18.3, 35.8)
Percentage of infant feedings that were breastmilk over first 6 months, median (range)	77.6 (55.1, 91.8)	98.0 (71.5, 100)	98.6 (91.4, 100)
Mother graduated from 4-year college	8 (40%)	15 (75%)	0
Maternal age in years at delivery, median (range)	28.5 (22.7, 37.9)	29.8 (25.1, 42.8)	21.5 (17.1, 32.1)
C-section delivery	16 (75%)	6 (30%)	11 (55%)

All comparisons significantly (p<0.05) different across the three sites, tested for continuous variables by Kruskal-Wallis test, and for categorical variables.

A total of 365 mother infant-pairs were recruited between all locations, of which 285 completed the study through 1 year, and milk samples from n = 60 donors (n = 20 from each location) were analyzed for carotenoid content. From our pilot data [2] we concluded that n = 20 would provide sufficient power to detect differences between lactation stages. Post-hoc power calculation indicates 73% power to detect a 50% relative difference in carotenoid content at a significance level of α = 0.05 when the relative standard deviation was approximately 60%. All mothers whose week 26 milk samples were used in this analysis were breastfeeding. Milk samples were collected between 9 AM and 1 PM using a Medela Symphony hospital grade breast pump. One entire breast was emptied of milk at the study visit in order to insure that foremilk, midmilk, and hindmilk were represented in the sample. Mothers were instructed to avoid feeding from the breast to be used for the sample two hours before the study visit. The contents were transported cold using gel packs and coolers for home visits, or refrigerated at the onsite visits until they were prepared for cryogenic storage, no later than 4 hours after pumping had occurred. Milk was thoroughly mixed by inverting the contents of the collection container to obtain a uniform suspension. For cryogenic storage, samples were aliquotted into barcoded, 2-ml cryogenic vials, screw cap with rubber gaskets (Sarstedt) using a polypropylene transfer pipette (Sarstedt), and frozen at -80°C.

Human milk samples were shipped in insulated boxes with adequate quantities of dry ice. All samples arrived frozen within 36 hours of shipment and were immediately stored at -80°C. Sample identification was blinded except for country of origin, and vials were assigned to a random order for analysis. Plasma samples of the mothers and infants in the USA cohort at 4 weeks postpartum were received from Cincinnati Children’s Hospital.

### Materials

Lutein, β-cryptoxanthin, β-carotene, lycopene, ammonium acetate, butylatedhydroxytoluene (BHT), sodium hydroxide, potassium hydroxide, L-ascorbic acid, Na_2_-ethylenediaminetetraacetic acid, ethyl gallate, and HPLC-grade denatured ethanol were from Sigma-Aldrich (St. Louis, MO). Solvents including ethyl acetate, methanol, isopropyl alcohol, acetone, petroleum ether, hexanes and HCl were purchased from Mallinckrodt Baker (Phillipsburg, NJ, USA). Echinenone, α-cryptoxanthin and α-carotene were from CaroteNature (Lupsingen, Switzerland). Zeaxanthin was from IndoFine (Hillsborough, NJ).

### Total lipid content

Total lipid content of human milk was determined in duplicate by the creamatocrit method developed by Lucas, et al. [21]. Intraday coefficient of variation (CV) determined by n = 4 analysis of the same human milk sample was 2.2%. Intraday CV determined across n = 3 days was 2.1%.

### Carotenoid extraction from milk

Carotenoids were extracted from human milk and infant formula by the method of Chauveua-Duriot, et al.[22] with slight modification. To minimize light and heat induced damage of carotenoids, extractions were performed in a room with yellow light shields and all solutions were placed on ice during extraction. Human milk (0.7mL) was diluted with 1.3 mL 0.9% NaCl, combined with 2 mL ethanol and 100 μL internal standard (50 nmol/L echinenone in ethanol), and shaken for 10 min. Lipids were extracted two times with 2 mL of 9:1 v/v hexanes:ethyl acetate with 0.1% w/v BHT. The solution was flushed with nitrogen, vortexed for 1 minute, shaken for 10 min, and centrifuged at 2,000xg for 5 min at 4°C before the organic layer was collected. Base-labile xanthophylls were extracted from the combined organic extracts twice with 2 mL 9:1 ethanol:water with 0.1% BHT in a similar manner, omitting shaking for 10 min. The combined ethanol layers were dried under a stream of nitrogen at 35°C then placed on ice. The residual organic extract was dried under nitrogen, saponified with 2 mL 10% w/v potassium hydroxide for 1 hour at 37°C, and then quenched with 2 mL chilled water. The solution was re-extracted three times with 2 mL 9:1 hexanes:ethyl acetate with 0.1% BHT. The organic layers were combined with the ethanolic extract, dried, resolubilized in 50μL ethyl acetate and 50μL methanol, centrifuged at 14K rpm for 5 min, and transferred to an HPLC vial.

### Carotenoid extraction from plasma

**Fig 2:**
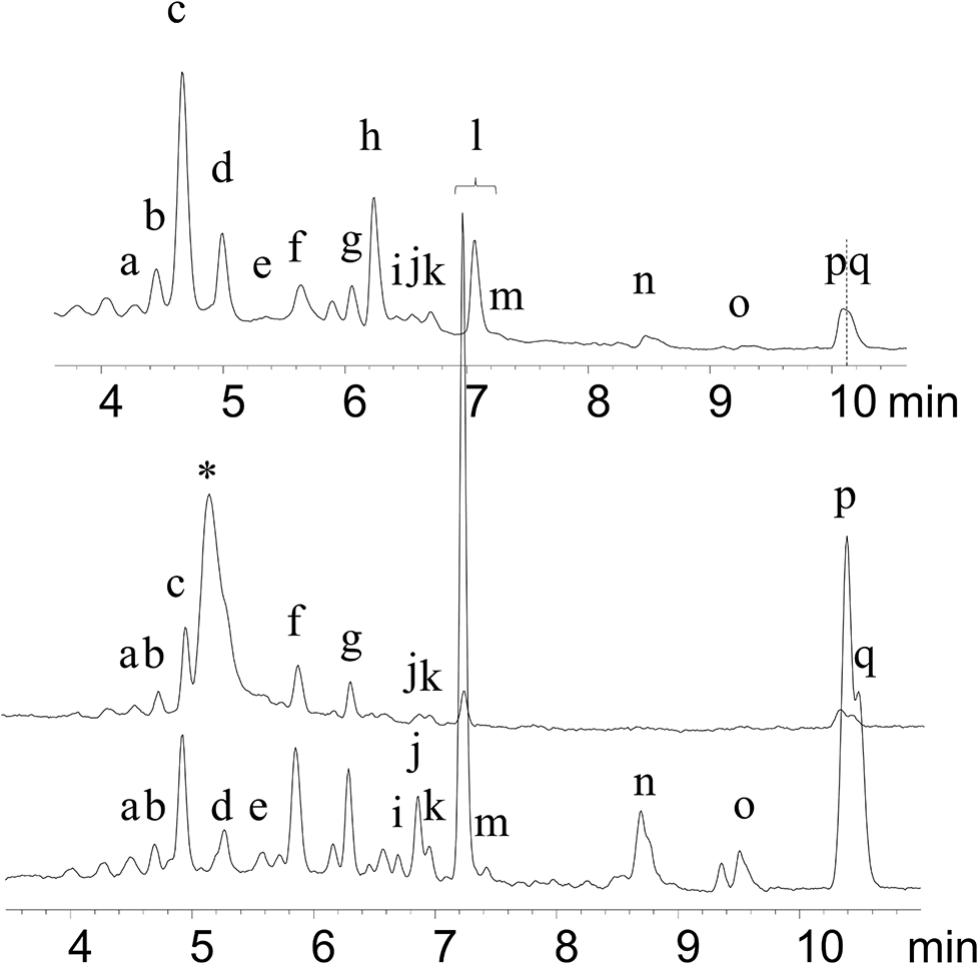
Chromatogram at 450 nm of carotenoids. Milk (top), neonatal plasma (middle), and maternal plasma (bottom) from the same family in the USA cohort at week 4. Peak identification: (a) *13*- or *13’-cis-*lutein (b) *13’-* or *13-cis-*lutein, (c) *all-trans-* lutein, (*) unidentified, (d) *all-trans*-zeaxanthin, (e) *9-* or *9’-cis-* lutein, (f) α-cryptoxanthin, (g) β-cryptoxanthin, (h) echinenone *internal standard*,(i) *15-cis*-β-carotene, (j) *13-cis*-β- carotene, (k) α-carotene, (l) *all-trans*-β- carotene, (m) *9-cis*-β-carotene, (n,o) *cis*-lycopene isomers, (p) *all-trans-*lycopene, (q) *5-cis-*lycopene.

Carotenoids were extracted from infant and maternal plasma in blinded, randomized order according to the method of Lipkie, et al. [23]. Briefly, 100 μL plasma was deproteinized with 250 μL methanol, and extracted 3 times with 1 mL 1:2 acetone/petroleum ether with 0.1% BHT. Recovery of echinenone internal standard averaged 95.3%. An unidentified peak unresolved from *all-trans*-zeaxanthin was observed in neonatal plasma (Fig 2) but not in maternal plasma or milk. As a result, zeaxanthin was not quantifiable from neonatal plasma. The identity could possibly be a zeaxanthin isomer, or a non-carotenoid lipid.

### Carotenoid quantification

Carotenoids were separated and quantified with an HP1090 HPLC as described previously [24] with diode array detection at 450 nm (Fig 2). A C30 2.0 x150 mm column from YMC (Kyoto, Japan) was used with a gradient of methanol with 2 mM ammonium acetate and ethyl acetate. Calibration curves were prepared spectrophotometrically with authentic *all-trans-* standards. *Cis-*lutein isomers were identified by diode array spectra and retention times from previous studies [25, 26] as well as iodine isomerization of *all-trans*-lutein standard [27], and quantified using the corresponding *all-trans-* standard response curve. Limit of quantitation for carotenoids was 0.1 pmol on column or 0.6 nmol/L in milk. Carotenoid content was corrected by the extraction efficiency of echinenone from each individual sample (average 68.4%, range 47.2–98.4%). Analytical repeatability was determined by repeat analysis of aliquots from the same sample (single donor and timepoint). Intraday coefficient of variation (CV) determined by n = 4 analysis of the same human milk sample was 5.4% and 7.0% for *all-trans-*β-carotene and *all-trans*-lutein, respectively. Interday CV determined across n = 3 days was 2.7% and 8.8% for *all-trans-*β-carotene and *all-trans*-lutein, respectively.

### Statistical analysis

Data were analyzed using SAS 9.3 (SAS Institute, Cary, NC). Due to the skewness and non-normality of carotenoid concentrations, transformations were identified using the Box-Cox procedure: carotenoid concentrations (nmol/L) were transformed as y’ = ln (y), expect for lycopene and total carotenoids, which were transformed as y’ = y^0.25^_,_ and total lipids as y’ = y^0.5^. Analysis of variance (ANOVA) for each transformed variable was completed using a factorial model of country (fixed categorical variable), week (continuous variable, i.e. linear regression), donor nested within country (blocking variable to increase power and to test for consistency within donors across time), and country by week interaction. All data presented in tables and figures are untransformed. P values represent type III sum of squares (with all other variables in the model). Superscripts denote significant differences between countries (α = 0.05) with all other variables in the model, with superscript a (^a^) denoting the country with the highest concentration. Box plots represent median, interquartile range (IQR), 25^th^ percentile—1.5 x IQR (or minimum, whichever is closer to the median), and 75^th^ percentile + 1.5 x IQR (or maximum, whichever is closer to the median). Carotenoid content of human milk (normalized per g lipid) was correlated to that of maternal plasma and neonatal plasma by simple linear regression.

## Results

### Carotenoid profile per volume

**Fig 3:**
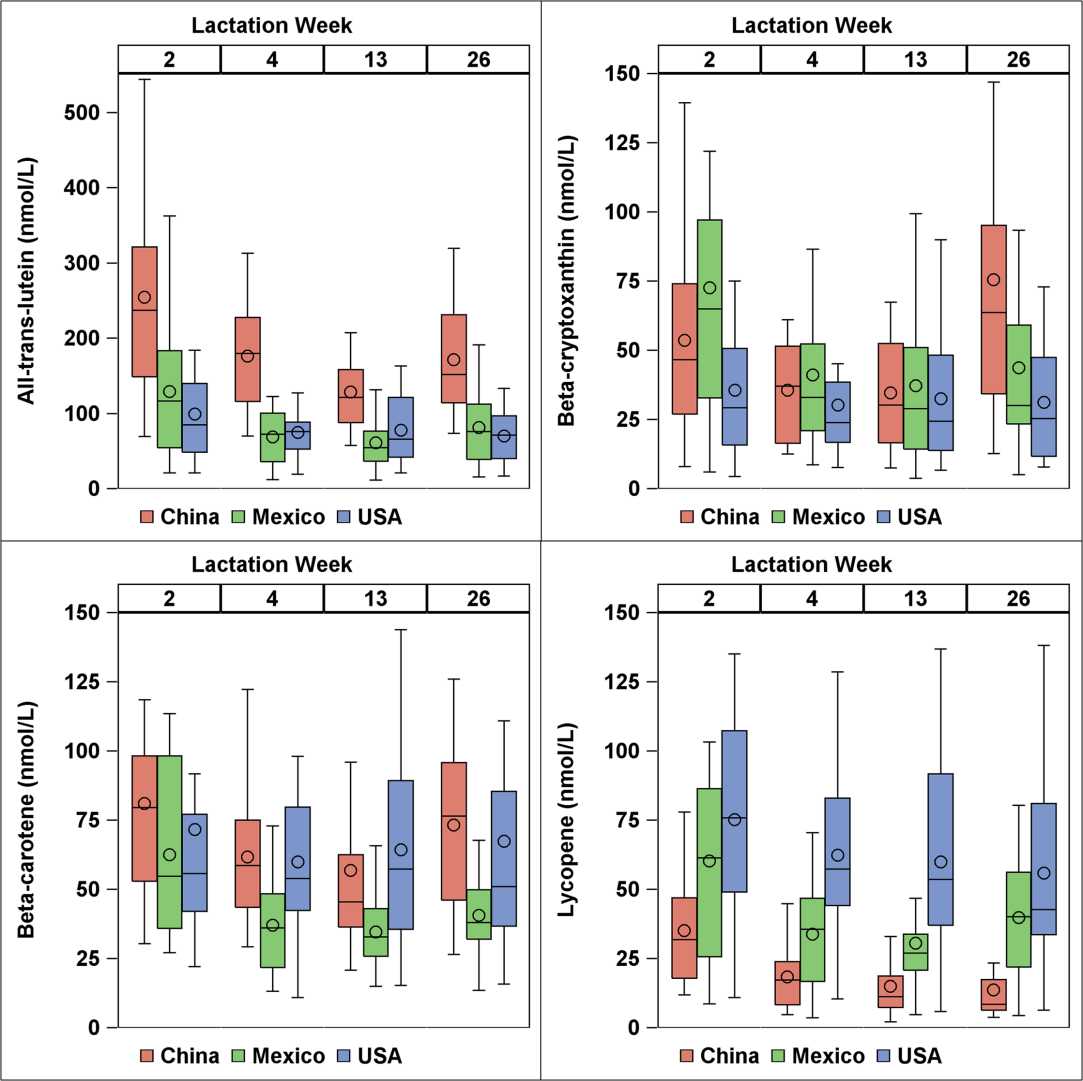
Box plots of major carotenoid species (sum of all identified cis and trans isomers) contents (nmol/L) by country and lactation stage. Boxes represent 25^th^, 50^th^, and 75^th^ percentiles. Circle represent mean. Whiskers represent either minimum/maximum or 25^th^/75^th^ minus/plus 1.5 x interquartile range, whichever is closer to the median.

Summary data of major carotenoid species in human milk across the three countries at all lactation stages are shown in Table 2.The complete data set for individual carotenoid concentrations in all 240 samples is in Table A in S1 File, and a summary by week and lactation stage is in Table B in S1 File. Box plots grouped by country and lactation stage for select carotenoids are shown in Fig 3. Median total carotenoid content of human milk across all countries and lactation stages was 328.5 nmol/L (interquartile range 217.9–463.0 nmol/L). ANOVA model of country, week, donor, and the country by week interaction indicated that carotenoid content on a volume basis decreased with lactation stage (p<0.05), except for the two provitamin A species β-cryptoxanthin and β-carotene (Table 2). Carotenoid content was strongly associated with donor (p<0.01 for all individual carotenoids). The observation that “donor” was a significant predictor (i.e. consistently trended within donors across lactation stages) suggests milk reflects chronic dietary exposure to carotenoids, and not a fleeting representation of acute carotenoid intake. Lutein and total carotenoids per volume were greatest (p<0.05) from China. Lycopene content was greatest (p<0.0001) in samples from the USA and lowest from China. Across all countries and lactation stages, the top four carotenoids were lutein (median 114.4 nmol/L), β-carotene (49.4 nmol/L), β-cryptoxanthin (33.8 nmol/L), and lycopene (33.7 nmol/L).

**Table 2:** Summary of carotenoid content of human milk and p-values of Country and Lactation stage ANOVA main effects (n = 60 donors, n = 240 samples).

	25th percentile	Median	75th percentile	Country^2^ (p-value)	Lactation Stage (p-value)
total lipids (g/L)	18.4	26.7	35.9	China^a^ Mexico^b^ USA^ab^ (p = 0.001)	Decrease (p = 0.0003)
lutein (nM)	70.0	114.4	179.5	China^a^ Mexico^b^ USA^b^ (p<0.0001)	Decrease (p = 0.0011)
zeaxanthin (nM)	20.5	30.1	48.1	China^a^ Mexico^ab^ USA^b^ (p = 0.003)	Decrease (p = 0.003)
α-cryptoxanthin (nM)	15.4	22.0	31.6	China^a^ Mexico^b^ USA^b^ (p = 0.001)	Not significant (p = 0.06)
β-cryptoxanthin (nM)	18.1	33.8	57.1	China^a^ Mexico^b^ USA^b^ (p = 0.02)	Not significant (p = 0.96)
α-carotene (nM)	8.4	13.7	20.3	China^a^ Mexico^a^ USA^a^ (p = 0.23)	Decrease (p = 0.04)
β-carotene (nM)	35.2	49.4	73.0	China^a^ Mexico^b^ USA^a^ (p<0.0001)	Not significant (p = 0.24)
lycopene (nM)	15.6	33.7	59.3	China^c^ Mexico^b^ USA^a^ (p<0.0001)	Decrease (p<0.0001)
total carotenoids (nM)	217.9	328.5	463.0	China^a^ Mexico^c^ USA^b^ (p<0.0001)	Decrease (p<0.0062)

Significant pairwise comparisons between countries are indicated by different superscripts

### Carotenoid profile per lipid basis

**Fig 4:**
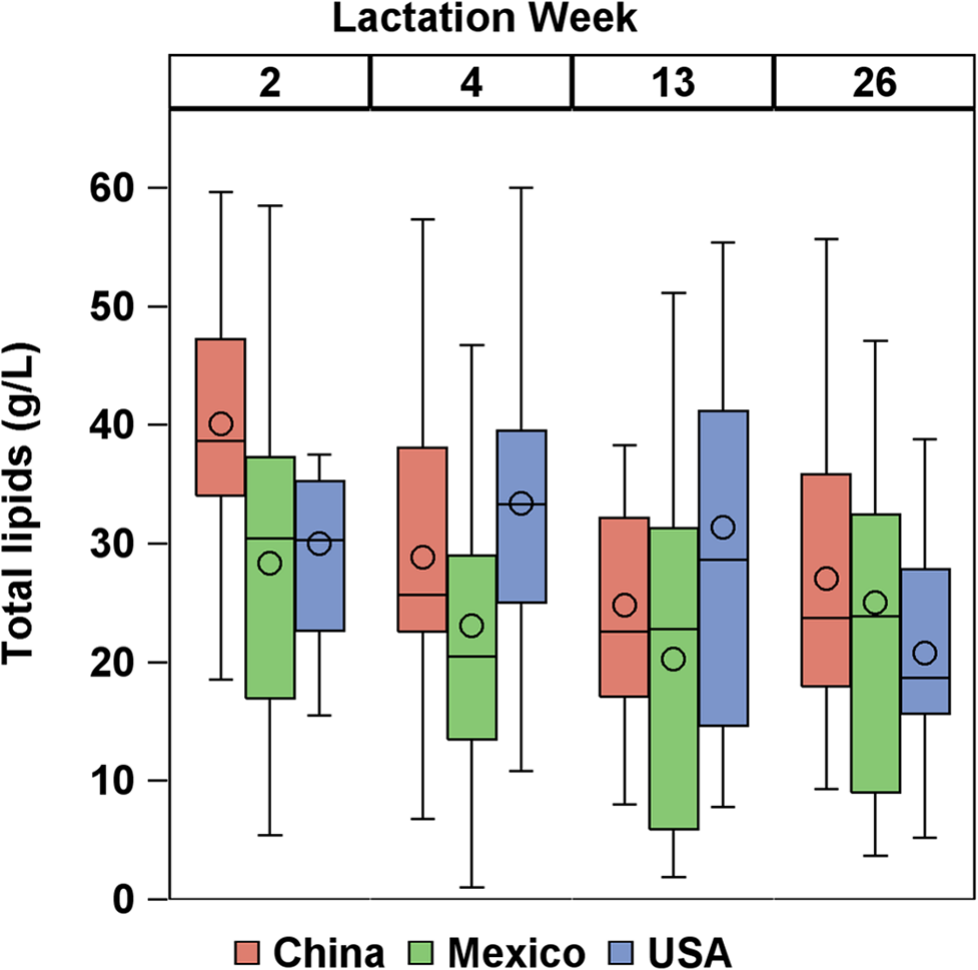
Total lipid content (g/L) by country and lactation stage. Boxes represent 25^th^, 50^th^, and 75^th^ percentiles. Circle represent mean. Whiskers represent either minimum/maximum or 25^th^/75^th^ minus/plus 1.5 x interquartile range, whichever is closer to the median.

Since carotenoids fluctuate with total lipids to some extent, ANOVA of carotenoid concentrations normalized by total lipid content are shown in Table C in S2 File. Total lipids by the creamatocrit method were greater (p = 0.001) from China (29.3 g/L) than from Mexico (23.7 g/L), and USA was not significantly different from the other two countries (28.8 g/L). Due to the low lipid content of milk from Mexican donors, carotenoids per g lipid were higher from China and Mexico than the USA (p<0.05). Total lipids significantly decreased with lactation stage (p = 0.002) (Fig 4). As a result, most carotenoids were not associated with lactation stage when reported on a per g lipid basis (see ANOVA analysis in Table C in S2 File). However, concentrations of provitamin A carotenoids α-cryptoxanthin, β-cryptoxanthin, and β-carotene with respect to total lipid increased (p<0.05) with increasing lactation stage.

### Plasma carotenoid associations

**Fig 5:**
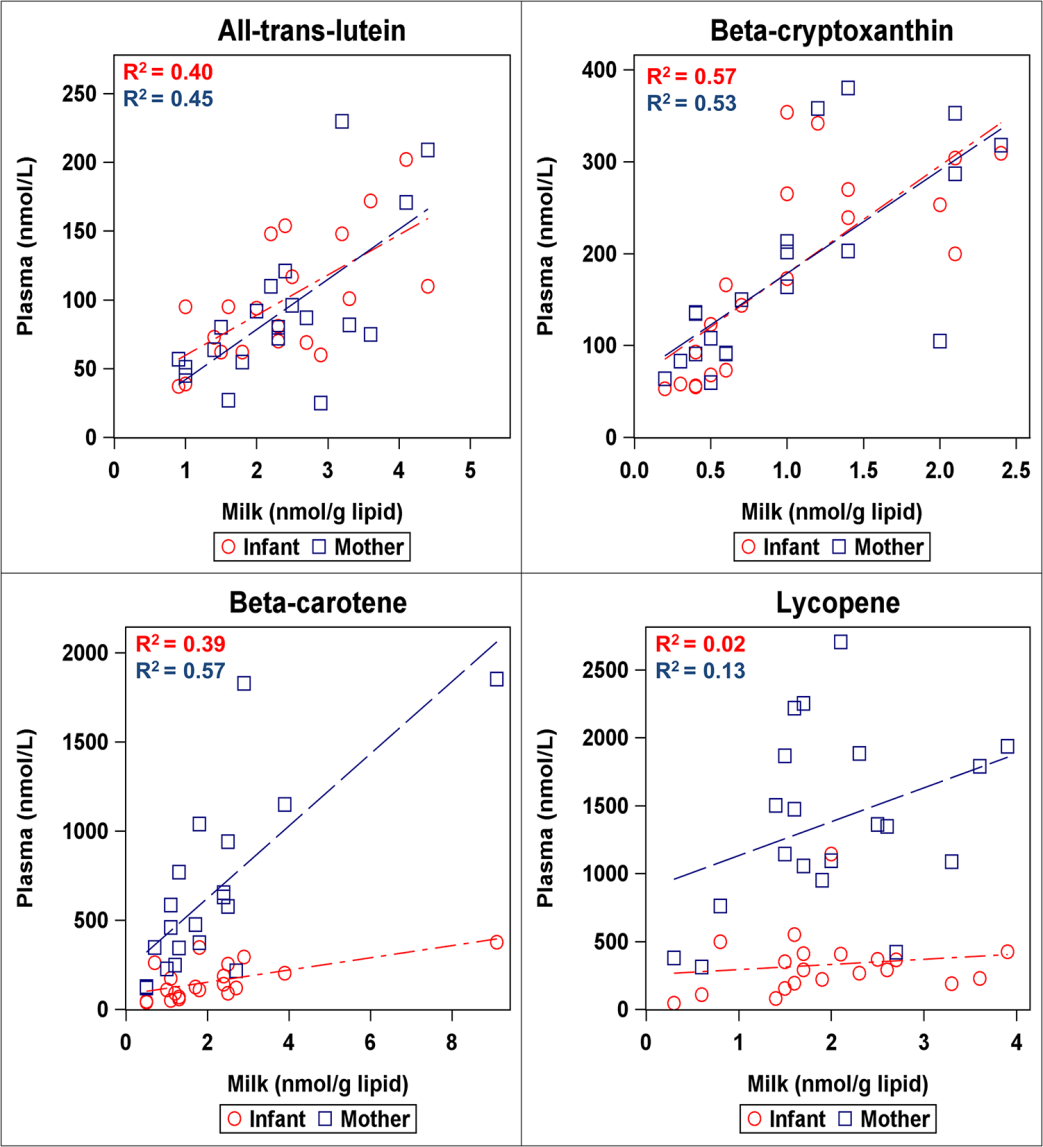
Scatterplots representing correlations between major carotenoids in milk (nmol/g lipid) and maternal/ neonatal plasma (nmol/L). Beta-carotene and lycopene represent sum of cis and trans isomers. All-trans-lutein was used for correlations instead of sum of isomers because cis-lutein isomers were below the detection limit in neonatal plasma.

Transfer of major carotenoids from maternal plasma to milk, milk to neonatal plasma, and overall from maternal plasma to neonatal plasma are depicted by scatterplots and correlations in Fig 5, with concentrations of other minor carotenoids separated by cis and trans isomers are reported in Table D in S2 File and correlations between maternal plasma, milk, and neonatal plasma in Table E in S2 File. Since cis-lutein isomers were below the detection limit in neonatal plasma (due to low concentration and small samples size of neonatal plasma), those correlations were derived from only *all-trans*-lutein. Correlations for all carotenoids except lycopene tended to be stronger when milk was represented on a lipid basis instead of a volume basis, so the following correlations will refer to milk concentrations in nmol/g lipid. *All-trans*-lutein, α-cryptoxanthin, and β-cryptoxanthin concentrations were strongly correlated (p<0.01) from maternal plasma to milk to neonatal plasma, *and* the average concentrations of these carotenoids were nearly equal between neonatal and maternal plasma. *All-trans-*β-carotene was also strongly correlated between maternal to milk to neonatal plasma (p = 0.004, R^2^ = 0.39), yet the average neonatal plasma concentration was about 4.5x lower than maternal concentration. Lycopene was significantly correlated between maternal plasma and milk (p<0.05), yet not between milk and neonatal plasma (p>0.3).

## Discussion

Carotenoid profiles of human milk followed similar trends from donors in China, Mexico, and the USA, despite distinct dietary patterns. We tend to report higher carotenoid levels than those reported by Canfield, et al. [18] for American, Chinese, and Mexican donors. Lutein was major carotenoid in human milk from all countries, which is in disagreement with a previous multinational study by Canfield, et al. [18] that found β-carotene and β-cryptoxanthin to be the major carotenoids in China and Mexico, respectively. This may be attributable to differences in several factors, such as time of collection (morning in the current study, afternoon in the previous [18]), method of milk expression, extraction methodology, subjects, and dietary patterns. Another study of milk from donors in Northern Ireland [28] also found a very similar concentration of lutein in human milk (median 4.79 nmol/g), but higher levels of most other carotenoids such as β-carotene (median 12.8 nmol/g). However, we did observe similar trends for particular carotenoids with increased prominence from individual countries, such as β-cryptoxanthin in Mexico (likely from papaya and orange), lycopene in Mexico and USA (likely from tomato), and lutein in China (likely from green vegetables).

As lactation stage proceeded, total carotenoid content decreased, especially from week 2 to week 4. This is in agreement with previous studies that report carotenoid content per volume decreases from parturition to 4 weeks postpartum [2, 13, 28] and stabilizes from 4–16 weeks postpartum [11, 29]. Total lipid content also decreased with lactation stage, which disagrees with previous reports that total lipid content increases steadily from parturition to 6 months postpartum [13, 30, 31]. Since both carotenoid content per volume and total lipid content decreased with lactation stage in the present study, total carotenoid content per lipid basis remained steady. Jewell, et al. [28] reported carotenoid content per lipid basis was highest at parturition then dropped quickly, stabilizing by week 2 to week 4. Our previous study [2] also observed a drop from week 1 to week 4 with carotenoids both per volume and per lipid basis. Since our sampling began at week 2, it may be that carotenoid content per lipid basis stabilized before longitudinal sampling began. In contrast to total and non-provitamin A carotenoids, provitamin A carotenoids were stable on a volume basis with lactation stage (increased per lipid basis). This physiological cause for this is unclear.

*Cis-*lutein isomers seem to be present in human milk at a roughly 1:3 ratio with *all-trans-*lutein. Presence of *cis-*lutein and zeaxanthin isomers have been identified before in human milk and plasma [32], as well as human and primate retina [33, 34]. Some isomers may originate from dietary sources [32] while some proportion may be isomerized from *all-trans-* species *in vivo*. Previous surveys of carotenoids across countries and lactation stages have not reported *cis* isomers, mostly likely due to lack of sufficient chromatographic separation of peaks. Depending on the chromatographic methodology *cis* isomers may be either uncounted or quantified with *all-trans-* isomers if the species were not resolved. *Cis-* isomers of lycopene were more prominent than *all-trans-lycopene* at a roughly 2:1 ratio in maternal plasma, neonatal plasma, and milk. This is consistent with previous observations that *cis*- isomers account for the majority of total lycopene in plasma and biological tissues but less than 10–25% in most foods such as unprocessed standard red tomatoes [27].

While carotenoid content was expressed per volume and per lipid basis to facilitate comparisons to the literature, data from this study suggests that milk carotenoid concentration per mass lipid may best reflect transfer of carotenoids from mother to infant. Correlations of human milk with both maternal and neonatal plasma were stronger when milk carotenoid content was expressed in units of nmol/g lipid than in nmol/L. Previous studies indicate that carotenoid concentration follows the total lipid content of breast milk within a feeding as the breast is emptied, i.e. both increase from foremilk to hindmilk [11, 12]. Even though carotenoids are associated with lipoproteins in circulation, once transferred to milk they are associated with the milkfat globule, and concentrations of carotenoids per total fat likely better reflect the secretion of carotenoids into the mammary gland. This would suggest that the total dietary intake of carotenoids by infants depends on the total intake of fat more than total volume of milk intake. However, since neonatal intake of human milk is often estimated on a volume basis, both measures may prove useful.

Neonatal plasma profile was similar to maternal plasma for xanthophylls (lutein, zeaxanthin, α-cryptoxanthin, and β-cryptoxanthin), but lower in the aliphatic carotenoids (α-carotene, β-carotene, and lycopene). Clinical, in vitro, and cell culture studies support the notion that polar xanthophylls are more bioavailable than carotenes, followed by lycopene [35]. Increased polarity may facilitate bioavailability by localizing xanthophylls at the surface of lipid structures, thereby enhancing transfer in and out of mixed micelles, lipoproteins, and milk fat globules. Carotenoid polarity is consistent with correlations between maternal plasma and milk carotenoid composition: the ratio of carotenoids in milk (nmol/L) to maternal plasma (nmol/L) was roughly 80% for lutein, 17% for cryptoxanthins, 7–13% for carotenes, and 4–5% for lycopene. This may also reflect a preference of mammary alveolar epithelial cells for HDL over LDL. However, this trend did not hold when comparing the ratio of neonatal plasma to human milk composition: 133% for lutein, 560–600% for cryptoxanthins, 270–300% for carotenes, and 480–650% for lycopene. Rubin, et al. [36] similarly observed neonatal plasma levels in relation to carotenoid intake from human milk was greatest for lycopene, intermediate from β-carotene, and lowest for lutein. However, the seemingly low response of lutein in neonatal plasma from milk content may also reflect metabolism and tissue uptake (i.e. lutein and zeaxanthin deposition in retinal tissue).

Strengths of this study include the longitudinal sampling of the same donors to follow lactation stage. This design is more sensitive to trends in lactation stage than a larger and potentially more representative cross sectional sample of donors, each donating at only one lactation stage. All donors from each country lived within a reasonable distance of a single collection site for each country, which increased reliability of sample collection with the same protocol. Limitations of this study include a small sample size, with limited locations, and. Carotenoid profiles may reflect those of the particular location more than the country as a whole. The dietary pattern and resulting carotenoid profile may not necessarily reflect those in rural areas or geographically distinct cities within the same country. Plasma associations of carotenoid transfer were only derived from the Cincinnati, USA location at 4 weeks postpartum because the likelihood of exclusive breastfeeding was much greater than at 13 and 26 weeks. However, neonatal-maternal plasma associations may not reflect those of other locations and lactation stages.

## Conclusions

This work deepens our understanding of neonatal exposure to carotenoids during development. The carotenoid content of human breast milk is highly variable between subjects, even within a given location and lactation stage, which makes it difficult to detect trends between countries and lactation stages unless those trends are very large. In general, carotenoid content tended to decrease with lactation stage as total lipid content decreased. Some particular carotenoids, such as lutein and lycopene, showed distinct differences between countries. We also report that the carotenoid content of maternal plasma is correlated to that of breast milk, which is again correlated to neonatal plasma content. Further work is needed in this area to understand the biological impact of carotenoid exposure to infants, and this study presents typical breast milk contents for such research. The breast milk carotenoid contents observed here across multiple nationalities and lactation stages may help guide dietary recommendations and the design of human milk mimetics.

## Supporting Information

S1 File Dataset. Data set of carotenoid species concentrations (nmol/L) in 240 human milk samples from 60 donors at 4 lactation stages, and corresponding summary statistics (**Table A**). Mean (interquartile range) summary by country and lactation stage (nmol/L) (**Table B**).
(XLSX)

S2 File Supporting Statistics Tables. ANOVA table for carotenoid content on a mass of lipid basis (**Table C**). Carotenoid content of maternal plasma, neonatal plasma, and milk from the USA cohort at 4 weeks postpartum (**Table D**). Statistics for correlations between maternal plasma (nmol/L), milk (nmol/g), and neonatal plasma (nmol/L) carotenoid contents (**Table E**).
(DOCX)
