# Supplementary material for: Longitudinal Survey of Carotenoids in Human Milk from Urban Cohorts in China, Mexico, and the USA
Source: PLoS One. 2015 Jun 10;10(6):e0127729. doi: 10.1371/journal.pone.0127729 (PMC4465022; doi:10.1371/journal.pone.0127729)
Supplement: S2 File — ANOVA table for carotenoid content on a mass of lipid basis (Table C). Carotenoid content of maternal plasma, neonatal plasma, and milk from the USA cohort at 4 weeks postpartum (Table D). Statistics for correlations between maternal plasma (nmol/L), milk (nmol/g), and neonatal plasma (nmol/L) carotenoid contents (Table E). (DOCX) [file pone.0127729.s002.docx]

# Supporting Information

**Table C. ANOVA table for carotenoid content on a mass of lipid basis.**

| *lipid basis* | *p-value of variable* | | | | |
| --- | --- | --- | --- | --- | --- |
| carotenoid | **transformation** | **country** | **week** | **donor** | **country*week** |
| *cis*-lutein | y’ = ln(y) | <0.0001 | 0.87 | 0.0013 | 0.55 |
| *all-trans*-lutein | y’ = ln(y) | <0.0001 | 0.18 | <0.0001 | 0.22 |
| *all-trans*-zeaxanthin | y’ = ln(y) | <0.0001 | 0.32 | <0.0001 | 0.42 |
| α-cryptoxanthin | y’ = ln(y) | 0.02 | 0.01 | <0.0001 | 0.12 |
| β-cryptoxanthin | y’ = ln(y) | <0.0001 | 0.0005 | <0.0001 | 0.0015 |
| α-carotene | y’ = ln(y) | 0.0009 | 0.14 | <0.0001 | 0.047 |
| *cis*-β-carotene | y’ = y^-0.5^ | 0.002 | 0.01 | 0.002 | 0.08 |
| *all-trans*-β-carotene | y’ = ln(y) | 0.47 | 0.01 | <0.0001 | 0.04 |
| *cis*-lycopene | y’ = y^0.25^ | <0.0001 | 0.64 | <0.0001 | 0.03 |
| *all-trans*-lycopene | y’ = ln(y) | <0.0001 | 0.56 | <0.0001 | 0.01 |
| total carotenoids | **y’ = y^-0.25^** | <0.0001 | 0.04 | <0.0001 | 0.1 |
| total lipids | **y’ = y^0.5^** | **0.001** | **0.0003** | **0.0005** | **0.25** |

**Table D. Carotenoid content of maternal plasma, neonatal plasma, and milk from the USA cohort at 4 weeks postpartum.**

|  | maternal plasma (nmol/L) | | neonatal plasma (nmol/L) | |
| --- | --- | --- | --- | --- |
| Carotenoid | mean ± SD | (min, max) | mean ± SD | (min, max) |
| *cis*-lutein | 76.7 ± 32.0 | (27.1, 144.4) | 49.0 ± 19.8 | (19.7, 97.6) |
| *all-trans*-lutein | 91.5 ± 54.8 | (24.8, 230.2) | 99.6 ± 45.2 | (36.8, 202.0) |
| *all-trans*-zeaxanthin | 44.2 ± 21.9 | (14.3, 92.3) | nq* | nq |
| α-cryptoxanthin | 127.4 ± 43.8 | (58.7, 207.6) | 124.8 ± 42.4 | (60.4, 196.2) |
| β-cryptoxanthin | 179.7 ± 105.4 | (60.4, 380.1) | 179.8 ± 105.5 | (52.6, 353.7) |
| α-carotene | 134.0 ± 81.7 | (18.1, 298.1) | 48.9 ± 25.6 | (11.2, 112.0) |
| *cis*-β-carotene | 81.0 ± 45.8 | (27.4, 188.9) | 29.4 ± 14.2 | (8.6, 58.5) |
| *all-trans*-β-carotene | 568.8 ± 456.5 | (79.9, 1664.5) | 128.9 ± 92.3 | (20.4, 322.9) |
| *cis*-lycopene | 824.1 ± 438.2 | (193.0, 1671.0) | 207.5 ± 132.2 | (17.6, 612.4) |
| *all-trans*-lycopene | 553.7 ± 248.6 | (119.4, 1034.8) | 122.7 ± 113.3 | (27.0, 531.4) |
| total carotenoids | **2681.2 ± 1178.4** | **(859.1, 5136.2)** | **1013.5 ± 422.7** | **(356.8, 2089.4)** |
|  | **milk (nmol/L)** | | **milk (nmol/g lipid)** | |
| Carotenoid | mean ± SD | (min, max) | mean ± SD | (min, max) |
| *cis*-lutein | 27.9 ± 12.1 | (7.8, 59.9) | 0.9 ± 0.4 | (0.4, 2.1) |
| *all-trans*-lutein | 74.5 ± 33.0 | (18.9, 145.6) | 2.4 ± 1.0 | (0.9, 4.4) |
| *all-trans*-zeaxanthin | 30.2 ± 11.5 | (7.2, 53.5) | 1.0 ± 0.4 | (0.4, 2.0) |
| α-cryptoxanthin | 22.1 ± 8.3 | (10.1, 38.1) | 0.7 ± 0.3 | (0.3, 1.2) |
| β-cryptoxanthin | 30.3 ± 20.0 | (7.7, 85.3) | 1.0 ± 0.7 | (0.2, 2.4) |
| α-carotene | 17.9 ± 9.8 | (4.4, 44.9) | 0.6 ± 0.3 | (0.1, 1.2) |
| *cis*-β-carotene | 17.5 ± 6.1 | (4.8, 30.5) | 0.6 ± 0.3 | (0.2, 1.4) |
| *all-trans*-β-carotene | 42.4 ± 27.9 | (3.9, 111.0) | 1.5 ± 1.6 | (0.1, 7.7) |
| *cis*-lycopene | 43.5 ± 23.3 | (5.5, 91.7) | 1.4 ± 0.7 | (0.1, 3.0) |
| *all-trans*-lycopene | 18.8 ± 8.8 | (4.1, 38.2) | 0.6 ± 0.2 | (0.1, 1.1) |
| total carotenoids | **325.1 ± 128.5** | **(120.1, 594.1)** | **10.6 ± 5.0** | **(4.0, 24.7)** |

* not quantifiable due to unresolved, unidentified interference

**Table E. Statistics for correlations between maternal plasma (nmol/L), milk (nmol/g), and neonatal plasma (nmol/L) carotenoid contents**. Correlations to milk carotenoids per lipid basis (nmol/g) were stronger than those to milk carotenoids per volume basis (not shown).

| *milk nmol/g lipid* | maternal plasma to milk | | milk to neonatal plasma | | maternal to neonatal plasma | |
| --- | --- | --- | --- | --- | --- | --- |
| Carotenoid | R^2^ | p | R^2^ | p | R^2^ | p |
| *cis*-lutein | 0.24 | 0.03 | 0.40 | 0.003 | 0.25 | 0.025 |
| *all-trans*-lutein | 0.45 | 0.001 | 0.43 | 0.002 | 0.34 | 0.004 |
| *all-trans*-zeaxanthin | 0.40 | 0.003 | nq* | nq | nq | nq |
| α-cryptoxanthin | 0.08 | 0.23 | 0.23 | 0.03 | 0.21 | 0.043 |
| β-cryptoxanthin | 0.53 | <0.001 | 0.57 | <0.001 | 0.52 | <0.001 |
| α-carotene | 0.36 | 0.006 | 0.09 | 0.11 | 0.08 | 0.23 |
| *cis*-β-carotene | 0.24 | 0.03 | 0.15 | 0.088 | 0.30 | 0.013 |
| *all-trans*-β-carotene | 0.61 | <0.001 | 0.41 | 0.002 | 0.39 | 0.004 |
| *cis*-lycopene | 0.11 | 0.15 | 0.06 | 0.32 | 0.06 | 0.30 |
| *all-trans*-lycopene | 0.248 | 0.03 | 0.010 | 0.67 | <0.01 | 0.97 |
| total carotenoids | **0.42** | **0.03** | **0.21** | **0.04** | **0.096** | **0.18** |

*not quantifiable
